# Association between Alpha B-crystallin expression and prognosis in patients with solid tumors: A protocol for systematic review and meta-analysis

**DOI:** 10.1097/MD.0000000000024831

**Published:** 2021-02-19

**Authors:** Minglan Yang, Yufan Li, Feng Tian

**Affiliations:** Digestive System Department, Linyi Central Hospital, Yishui 276400, Shandong Province, China.

**Keywords:** alpha B-crystallin, meta-analysis, prognosis, solid tumor

## Abstract

**Background:**

Alpha B-crystallin (CRYAB), as a small heat shock protein, may play critical roles in the tumorigenesis and progression of several kinds of human cancers. However, the prognostic value of CRYAB in solid malignancies remains controversial. The aim of the present study was to investigate the association between CRYAB expression and clinicopathology and prognosis of solid tumor patients.

**Methods:**

PubMed, Web of Science, EMBASE, Chinese National Knowledge Infrastructure, and WanFang databases were systematically searched to retrieve studies that investigated the prognostic value of CRYAB expression in various solid tumors. Hazard ratios (HRs) with 95% confidence intervals (CIs) were calculated to determine the strength of association between CRYAB expression and survival in patients with solid tumors. Odds ratios (ORs) with 95% CIs were pooled to assess the correlation between CRYAB expression and clinicopathological characteristics of patients with solid tumors.

**Results:**

A total of 17 studies, including 18 cohorts with 6000 patients, were included in this meta-analysis. Our results showed that increased CRYAB expression could predict poor overall survival (HR = 1.81, 95% CI: 1.50–2.19, *P* < .001), disease-free survival (HR = 1.47, 95% CI: 1.16–1.86, *P* = .001), and disease-specific survival (HR = 1.40, 95% CI: 1.19–1.63, *P* < .001) in patients with cancer. Furthermore, the high expression level of CRYAB was associated with certain phenotypes of tumor aggressiveness, such as lymph node metastasis (OR = 2.46, 95% CI: 1.48–4.11, *P* = .001), distant metastasis (OR = 3.34, 95% CI: 1.96–5.70, *P* < .001), advanced clinical stage (OR = 2.24, 95% CI: 1.24–4.08, *P* = .008), low OS rate (OR = 4.81, 95% CI: 2.82–8.19, *P* < .001), and high recurrence rate (OR = 1.38, 95% CI: 1.11–1.72, *P* = .004).

**Conclusions:**

CRYAB may serve as a valuable prognostic biomarker and therapeutic target in human solid tumors.

## 1 Introduction

Epidemiological data show that cancer is a major cause of death in both developing and developed countries.^[1]^ According to latest data, solid cancers characterized by malignant tumors that form a discrete tumor mass accounted for more than 90% of all types of cancers.^[1]^ Although considerable progress has been made on targeted therapies and comprehensive treatments, the prognosis of the vast majority of patients remains poor.^[2]^ A lack of biomarkers for the early detection and precise diagnosis has limited the efficacy of current therapies for patients with solid tumors.^[3]^ Thus, identifying other predictive molecular markers of human solid tumors is of primary importance in improving therapy and prognosis.^[4]^

Alpha B-crystallin (CRYAB), also called HspB5, is a member of the small molecule heat shock protein family and was first discovered as a major structural protein in the lens of the eyes.^[5,6]^ CRYAB acts primarily as a molecular chaperone: when cells are exposed to external stress, such as heat shock, oxidative stress, radiation, and exposure to anticancer drugs, CRYAB binds to unfolded proteins, inhibits their aggregation, and prevents degeneration and degradation, thereby promoting cell survival, inhibiting apoptosis, protecting cells, and degrading proteases.^[7,8]^ In addition, CRYAB promotes tumor cell invasion and metastasis through epithelial-mesenchymal transition (EMT). Recent studies on CRYAB's role in tumorigenesis and progression have attracted attentions. Several publications have recently claimed that CRYAB overexpression is significantly associated with poor prognosis in various types of cancer, while other studies have reported that the connection is not significant.^[5–7,9–22]^ Therefore, we conducted this meta-analysis to evaluate the correlation between high CRYAB expression and the prognosis of human solid tumors, and to clarify the clinical value of CRYAB as a potential prognostic indicator and therapeutic target for human solid tumors.

## 2 Materials and methods

### 2.1 Ethics approval

This study is a meta-analysis of data from published articles and does not include human participants or animals. Therefore, ethics approval is not required for this study.

### 2.2 Search strategy

Two independent authors (YML and LYF) performed a comprehensive literature search of PubMed, Web of Science, EMBASE, Chinese National Knowledge Infrastructure, and WanFang databases to identify relevant studies on CRYAB expression and survival in patients with solid tumor prior to August 2020. The following terms were used in the search strategy: (“CRYAB” OR “Alpha B crystallin” OR “αB-crystallin” OR “crystallin αB” OR “HspB5”) AND (“tumor” OR “cancer” OR “carcinoma” OR “malignancy” OR “neoplasms”) AND (“survival” OR “outcome” OR “prognosis”). The references from the articles were also scanned to determine studies of possible interest. Any discrepancy was resolved by consensus through discussion.

### 2.3 Inclusion and exclusion criteria

We diligently selected the eligible articles based on the following inclusion criteria:

(1) patients were pathologically diagnosed with solid tumors;
(2) CRYAB expression was measured through immunohistochemistry stain in the tumor tissue;
(3) the correlation between CRYAB expression and prognostic outcomes, including overall survival (OS), disease-free survival (DFS), and disease-specific survival (DSS), was investigated; and
(4) hazard ratios (HRs) and 95% confidence intervals (CIs) can be directly extracted or calculated based on survival curves.

We excluded studies in accordance with the following criteria:

(1) letters, reviews, abstracts, case reports, comments, or animal experiments;
(2) studies without sufficient data to extract or estimate HRs and their 95% CIs;
(3) CRYAB expression was not divided into 2 groups: “high” and “low” or “positive” and “negative;” and
(4) studies with a sample size less than 50.

### 2.4 Data extraction and quality assessment

All data from eligible studies were reviewed and extracted independently by 2 authors (YML and LYF). The following data were collected: the first author, publication year, study country, cancer type, duration time, follow-up time, number of patients, detection method, cutoff value, number and percentage of high CRYAB expression, prognostic outcome, analysis method, HRs with 95% CIs of high CRYAB expression group compared with low expression group, and language and clinicopathological features. If HR values of univariate and multivariate analyses were both provided in the article, only the latter was chosen because it took confounding factors into account and was more accurate than the former.

The quality of each included article was assessed by 2 independent authors (YML and LYF) using the Newcastle-Ottawa Scale (NOS). The NOS scale comprises 3 evaluation contents: selection of the exposed and unexposed cohort, 0 to 4; comparability of the 2 cohorts, 0 to 2; and outcome assessment, 0 to 3.^[23]^ Each study received a consensus NOS score by discussion. Studies with a score ≥ 6 were considered as methodologically sound.

### 2.5 Statistical analysis

The HRs and 95% CIs were combined to assess the association between CRYAB expression and survival endpoints (OS, DFS, and DSS) in patients with malignant solid tumors. The odds ratios (ORs) and 95% CIs were pooled to evaluate the correlation between CRYAB expression and clinicopathological features. Heterogeneity assumption was evaluated by chi-based *Q*-test and *I*^2^ metric.^[24]^ The *P* value < .05 or the *I*^2^ value > 50% indicated significant heterogeneity. The random-effects model was used for studies with significant heterogeneity. Otherwise, a fixed-effects model was applied. Subgroup and meta-regression analyses were conducted to investigate the suspected factors for heterogeneity. Sensitivity analysis was performed by sequentially omitting individual study to verify the stability of the meta-analysis results. The effect of potential publication bias on prognosis was quantitatively evaluated using Begg and Egger asymmetry tests, and was visually evaluated by funnel plots.^[25]^ When significant publication bias existed, the trim-and-fill method was performed to appraise the robustness of analysis results. All calculations were conducted using the STATA version 12.0 software (Stata, College Station, TX). All statistical tests were 2-sided and *P* < .05 was deemed as statistically significant.

## 3 Results

### 3.1 Literature search and study demographics

**Figure 1:**
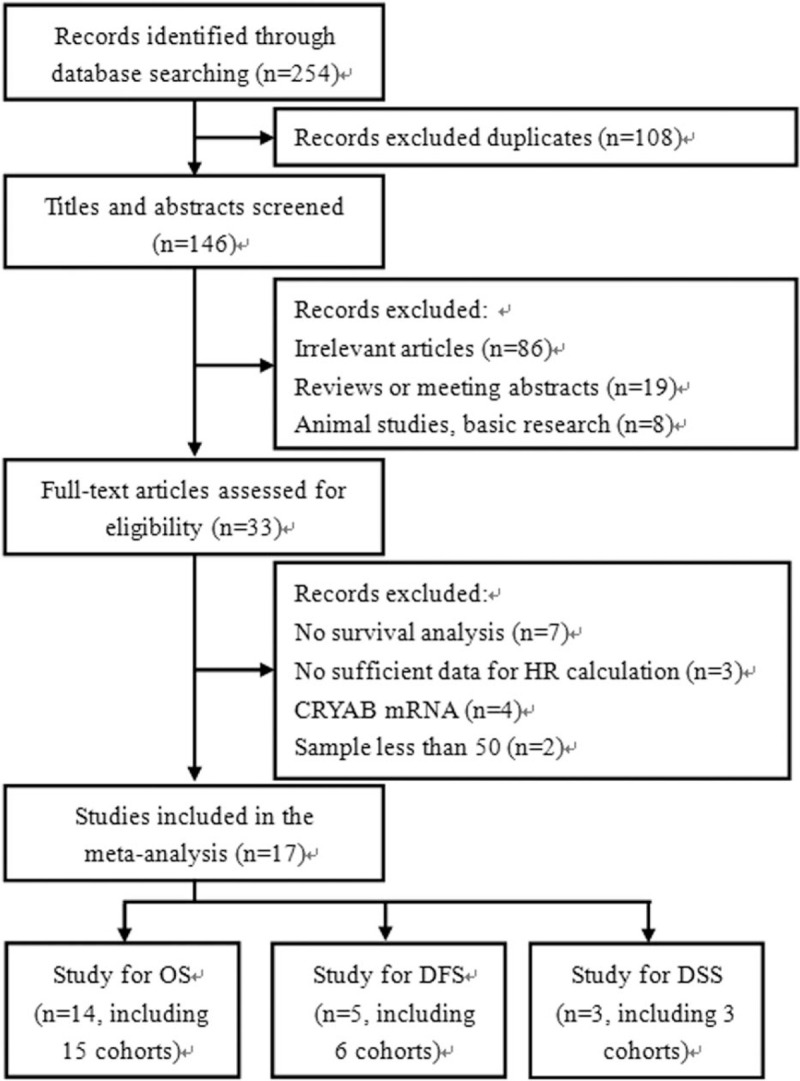
Flow diagram of the study selection process and specific reasons for exclusion in the meta-analysis.

A total of 254 reports were retrieved from a primary literature search, and 108 duplicate records were removed. After the titles and abstracts were screened, 113 papers were further excluded as follows: apparently irrelevant articles (n = 86), reviews or meeting abstracts (n = 19), and non-human studies (n = 8). As for the remaining 33 studies, the reasons for exclusion were as follows: 7 did not perform survival analysis, 3 did not provide sufficient data for HR calculation, 4 studied the association between CRYAB mRNA expression and prognosis, and 2 had a sample size less than 50. Finally, 17 articles with 18 cohorts published from 2006 to 2019 were included in the meta-analysis. The process of literature selection is shown in Figure 1.

The main characteristics of the included studies are summarized in Table 1. A total of 6000 patients from China,^[6,7,9–11,13,16,17,19,21]^ Korea,^[12,14,20]^ Canada,^[15]^ Sweden,^[5]^ Taiwan,^[18]^ and USA^[22]^ were diagnosed with distinct cancers, including breast cancer (BC),^[9,12,15,20,22]^ gastric cancer,^[6]^ ovarian cancer (OC),^[7]^ non-small cell lung cancer (NSCLC),^[10,17]^ laryngeal squamous cell carcinoma,^[11,19]^ colorectal cancer,^[13,16]^ renal cell carcinoma (RCC),^[14,18]^ oral or oropharyngeal squamous cell carcinoma,^[5]^ and hepatocellular carcinoma.^[21]^ The sample sizes ranged from 50 to 3987, and CRYAB expression was evaluated by immunohistochemistry in all studies. Fifteen cohorts provided data on OS,^[5–7,9–14,16–19,21]^ 6 cohorts provided data on DFS,^[7,12,13,15,20]^ and 3 cohorts provided data on DSS^[5,15,22]^ with respect to survival. Also, 17 HRs were obtained by multivariate analysis,^[6,7,9–11,13–19,21]^ while the remaining 7 HRs were calculated by univariate analysis.^[5,12,15,20,22]^ According to the NOS, all cohort studies had scores greater than or equal to 6 and were of relatively high quality.

**Table 1:** Main characteristics of the eligible studies.

Study	Region	Duration	Cancer type	Clinical stage	Follow up (months)	Number	Detection method	Cut-off value	CRYAB-high (%)	Survival analysis	Language	Quality
Zeng L 2019	China	1996-2005	BC	I-III	> 120	190	IHC	NR	51 (26.8)	OS (M)	English	8
Tao X 2019	China	2012-2013	GC	I-IV	Until Dec 2018	100	IHC	≥ 3	57 (57.0)	OS (M)	English	8
Tan L 2019 (E)	China	2004-2015	OC	I-IV	NR	103	IHC	NR	63 (61.2)	OS (M), DFS (M)	English	7
Tan L 2019 (V)	China	2004-2015	OC	I-IV	NR	103	IHC	NR	67 (65.0)	OS (M), DFS (M)	English	7
Gu J 2018	China	2005	NSCLC	I-IV	NR	208	IHC	> 3	106 (51.0)	OS (M)	Chinese	7
Xu L 2016	China	2000-2009	LSCC	I-IV	NR	80	IHC	> 4	42 (52.5)	OS (M)	Chinese	7
Zhu J 2015	China	2005-2008	CRC	I-IV	Until Dec 2012	100	IHC	≥ 25%	68 (68.0)	OS (M), DFS (M)	Chinese	8
Voduc KD 2015	Canada	1986-1992	BC	NR	Median 144	3987	IHC	> 0	359 (9.0)	DSS (M), DFS (U)	English	8/6
Kim MS 2015	Korea	2003-2012	RCC	I-IV	Mean 60	91	IHC	180	56 (61.5)	OS (M)	English	8
Kim MS 2015	Korea	2003-2009	IDC	I-III	Mean 84	82	IHC	> 0	18 (22.0)	OS (U), DFS (U)	English	6
Shi C 2014	China	2002-2007	CRC	I-IV	NR	100	IHC	≥ 3	58 (58.0)	OS (M)	English	7
Qin H 2014	China	2005-2006	NSCLC	I-IV	NR	101	IHC	≥ 4	45 (44.6)	OS (M)	English	7
Annertz K 2014	Sweden	1990-1999	OOPSCC	I-IV	Median 20	55	IHC	TQ	40 (72.7)	OS (U), DSS (U)	English	6
Ho P 2013	Taiwan	2002-2009	ccRCC	NR	Median 44.5	50	IHC	> 40	13 (26.0)	OS (M)	English	8
Mao Y 2012	China	2000-2009	LSCC	I-IV	60	109	IHC	≥ 4	64 (58.7)	OS (M)	English	8
Kim HS 2011	Korea	2002-2006	IDC	NR	Mean 50	82	IHC	≥ 2	30 (36.6)	DFS (U)	English	6
Tang Q 2009	China	2002-2005	HCC	I-IV	24-60	98	IHC	≥ 3	42 (42.9)	OS (M)	English	8
Moyano JV 2006	USA	1974-1995	IBC	NR	Medain 130	361	IHC	> 0	39 (10.8)	DSS (U)	English	6

### 3.2 Association between CRYAB expression and clinicopathological features

The correlations between CRYAB expression and clinicopathological parameters in human solid tumors are presented in Table 2. Thirteen cohorts with 1363 patients investigated the relationship between CRYAB expression and lymph node metastasis, and the pooled result showed that high CRYAB expression was associated with positive lymph node metastasis (OR = 2.46, 95% CI: 1.48–4.11, *P* = .001, random effects). Five cohorts with 462 cases reported the correlation between CRYAB expression and distant metastasis, and the conjoined result suggested that CRYAB overexpression was significantly related to positive distant metastasis (OR = 3.34, 95% CI: 1.96–5.70, *P* < .001, fixed effects). The association between CRYAB expression and clinical stage was evaluated in 11 cohorts with 1292 participants, and the pooled analysis demonstrated that high CRYAB expression was significantly associated with advanced clinical stage (OR = 2.24, 95% CI: 1.24–4.08, *P* = .008, random effects). Moreover, 6 cohorts focused on the correlation between CRYAB expression and OS, and found that positive CRYAB expression was correlated with low OS rate (OR = 4.81, 95% CI: 2.82–8.19, *P* < .001, random effects). Furthermore, pooled analysis of 3 cohorts demonstrated that high expression of CRYAB was significantly associated with high recurrence rate (OR = 1.38, 95% CI: 1.11–1.72, *P* = .004, fixed effects). However, the association between CRYAB expression and age (OR = 0.99, 95% CI: 0.78–1.26, *P* = .945, fixed effects), gender (OR = 1.12, 95% CI: 0.82–1.53, *P* = .478, fixed effects), tobacco use (OR = 0.78, 95% CI: 0.54–1.14, *P* = .196, fixed effects), and depth of invasion (OR = 1.23, 95% CI: 0.18–8.53, *P* = .832, random effects) was not significant.

**Table 2:** Meta-analysis of CRYAB and clinicopathological features in cancer patients.

Categories	Trials (Patients)	OR (95%CI)	*I*^2^ (%)	*P*_*h*_	*Z*	*P*_*z*_
Age (young vs old)	11 (1292)	0.99 (0.78–1.26)^F^	0.0	.756	0.07	.945
Gender (male vs female)	9 (987)	1.12 (0.82–1.53)^F^	11.6	.338	0.71	.478
Tobacco use (no vs yes)	4 (498)	0.78 (0.54–1.14)^F^	0.0	.683	1.29	.196
Depth of invasion (T1-T2 vs T3-T4)	3 (273)	1.23 (0.18–8.53)	86.6	.001	0.21	.832
Lymph node metastasis (negative vs positive)	13 (1363)	2.46 (1.48–4.11)	71.3	<.001	3.46	.001
Distant metastasis (negative vs positive)	5 (462)	3.34 (1.96–5.70)^F^	36.9	.175	4.44	<.001
clinical stage (I-II vs III-IV)	11 (1292)	2.24 (1.24–4.08)	80.6	<.001	2.65	.008
Overall survival (alive vs dead)	6 (596)	4.81 (2.82–8.19)	51.1	.069	5.77	<.001
Recurrence (negative vs positive)	3 (3391)	1.38 (1.11–1.72)^F^	3.0	.357	2.87	.004

### 3.3 Association between CRYAB expression and prognosis

**Figure 2:**
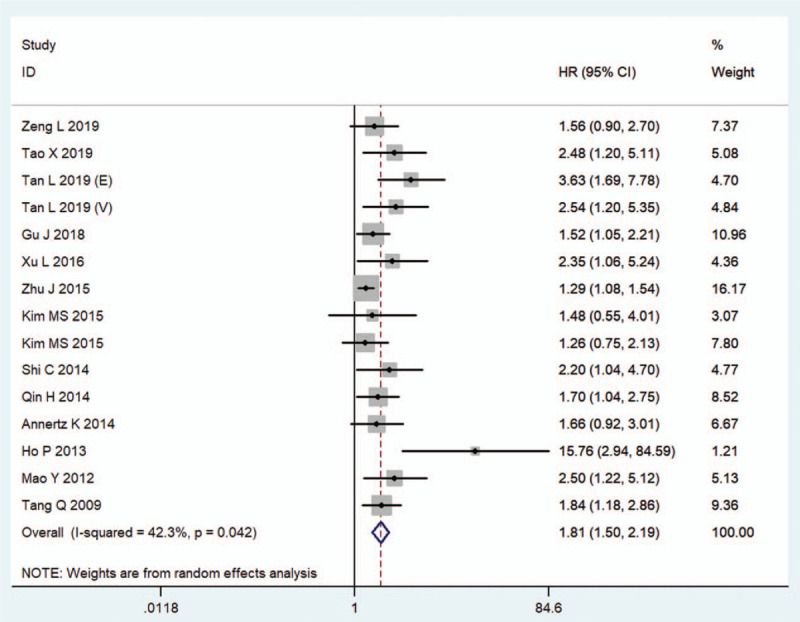
Forest plots of the overall outcomes for overall survival. CI = confidence interval, HR = hazard ratio.

The main meta-analysis results of the relationship between CRYAB expression and the prognosis of patients with solid tumors are shown in Table 3. Fifteen cohorts, with 1570 patients reported the HRs for OS. The random-effects model was applied to estimate the combined HR and 95% CI because of significant heterogeneity (*I*^*2*^ = 42.3%, *P* = .042). The pooled results showed that positive CRYAB expression was closely associated with poor OS (HR = 1.81, 95% CI: 1.50–2.19, *P* < .001) (Fig. 2). When subgroup analysis was conducted on the basis of cancer type, increased CRYAB expression was significantly associated with unfavorable OS in patients with digestive system cancers (HR = 1.43, 95% CI: 1.22–1.67, *P* < .001), head and neck cancer (HR = 2.05, 95% CI: 1.38–3.05, *P* < .001), OC (HR = 3.03, 95% CI: 1.77–5.16, *P* < .001), NSCLC (HR = 1.58, 95% CI: 1.18–2.13, *P* = .002), but not in patients with BC (HR = 1.40, 95% CI: 0.96–2.04, *P* = .084), RCC (HR = 4.37, 95% CI: 0.43–43.97, *P* = .211). Subgroup analysis by clinical stage suggested that CRYAB overexpression had an adverse effect on OS for patients with Stage I-IV (HR = 1.58, 95% CI: 1.39–1.79, *P* < .001), none reported (HR = 15.76, 95% CI: 2.94–84.59, *P* = .001), but not for patients with stage I-III (HR = 1.40, 95% CI: 0.96–2.04, *P* = .084). In subgroup analysis based on the sample size, the result showed that high CRYAB expression had significantly poor OS in both large (HR = 1.53, 95% CI: 1.34–1.75, *P* < .001) and small (HR = 1.75, 95% CI: 1.34–2.27, *P* < .001) sample sizes. Subgroup analysis according to the proportion of patients with high CRYAB expression showed that CRYAB overexpression was closely related to shorter OS in the high (HR = 1.54, 95% CI: 1.34–1.77, *P* < .001) and low (HR = 1.75, 95% CI: 1.21–2.55, *P* = .003) proportions. With regard to the analysis method, CRYAB positive expression predicted short OS in multivariate analysis (HR = 1.91, 95% CI: 1.54–2.38, *P* < .001), but not in univariate analysis (HR = 1.42, 95% CI: 0.96–2.11, *P* = .078). Therefore, the results did not markedly change when subgroup analyses were performed according to cancer type, clinical stage, sample size, proportion of patients with high CRYAB expression, and analysis method. Furthermore, meta-regression analysis demonstrated that cancer type (*P* = .698), clinical stage (*P* = .267), sample size (*P* = .393) proportion of patients with high CRYAB expression (*P* = .306), and analysis method (*P* = .174) were not sources of heterogeneity for OS.

**Table 3:** Summary of the meta-analysis results.

Categories	Trials (patients)	HR (95%CI)	*I*^2^ (%)	*P*_*h*_	*Z*	*P*_*z*_	*P*_*m*_
OS (All)	15 (1570)	1.81 (1.50–2.19)	42.3	.042	6.10	<.001	
Cancer type							.698
Digestive system	4 (398)	1.43 (1.22–1.67)^F^	49.6	.114	4.39	<.001	
HNC	3 (244)	2.05 (1.38–3.05)^F^	0.0	.642	3.54	<.001	
BC	2 (272)	1.40 (0.96–2.04)^F^	0.0	.582	1.73	.084	
OC	2 (206)	3.03 (1.77–5.16)^F^	0.0	.512	4.07	<.001	
NSCLC	2 (309)	1.58 (1.18–2.13)^F^	0.0	.720	3.06	.002	
RCC	2 (141)	4.37 (0.43–43.97)	82.3	.018	1.25	.211	
Clinical stage							.267
Stage I-IV	12 (1248)	1.58 (1.39–1.79)^F^	32.8	.128	7.02	<.001	
Stage I-III	2 (272)	1.40 (0.96–2.04)^F^	0.0	.582	1.73	.084	
NR	1 (50)	15.76 (2.94–84.59)	-	-	3.22	.001	
Sample size							.393
≥100	9 (1114)	1.53 (1.34–1.75)^F^	45.7	.064	6.20	<.001	
<100	6 (456)	1.75 (1.34–2.27)^F^	43.1	.118	4.16	<.001	
CRYAB-high (%)							.306
≥50%	10 (1049)	1.54 (1.34–1.77)^F^	42.7	.073	6.17	<.001	
<50%	5 (521)	1.75 (1.21–2.55)	51.2	.085	2.95	.003	
Analysis method							.174
Multivariate	13 (1433)	1.91 (1.54–2.38)	49.0	.024	5.78	<.001	
Univariate	2 (137)	1.42 (0.96–2.11)^F^	0.0	.497	1.76	.078	
DFS (All)	6 (4457)	1.47 (1.16–1.86)	62.6	.020	3.19	.001	
DSS (All)	3 (4403)	1.40 (1.19–1.63)^F^	1.0	.364	4.15	<.001	

**Figure 3:**
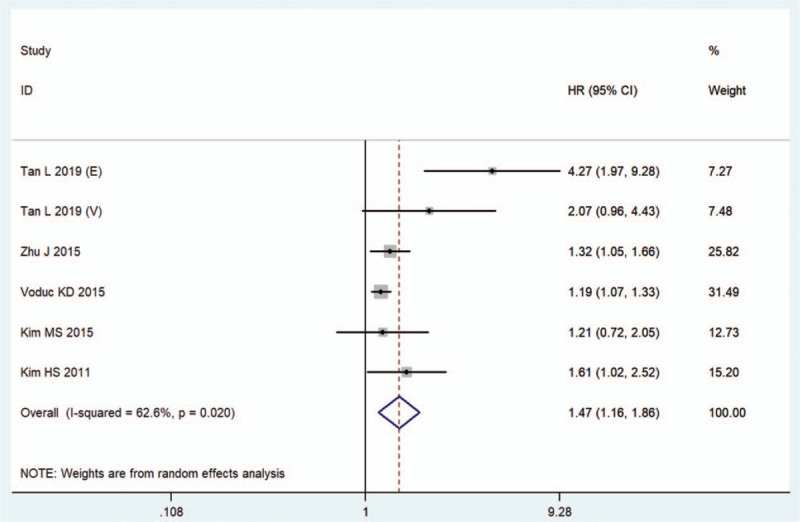
Forest plots of the overall outcomes for disease-free survival. CI = confidence interval, HR = hazard ratio.

**Figure 4:**
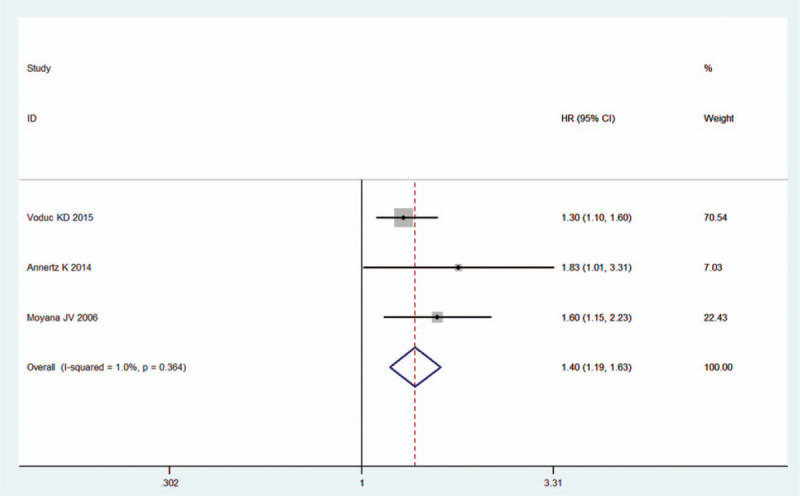
Forest plots of the overall outcomes for disease-specific survival. CI = confidence interval, HR = hazard ratio.

Additionally, 6 cohorts comprising 4457 participants reported the survival endpoint of DFS, and the combined result revealed that increased CRYAB expression was predictive of reduced DFS (HR = 1.47, 95% CI: 1.16–1.86, random effect; Table 3, Fig. 3) with significant heterogeneity (*I*^*2*^ = 62.6%, *P* = .020). DSS was studied in 3 cohorts, including 4403 patients. The meta-analysis showed that high CRYAB expression in tumor tissues significantly increased the risk of shortening DSS (HR = 1.40, 95% CI: 1.19–1.63, fixed effect; Table 3, Fig. 4).

### 3.4 Sensitivity analysis and publication bias

**Figure 5:**
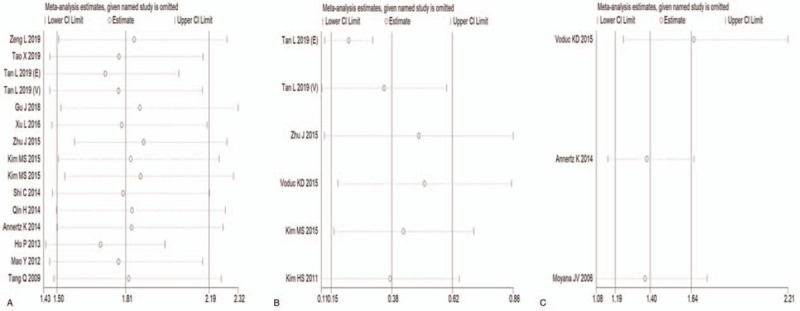
Effects of individual studies on pooled hazard ratios for CRYAB and survival in solid tumors. (A) Result of sensitivity analysis for pooled overall survival estimation. (B) Result of sensitivity analysis for pooled disease-free survival estimation. (C) Result of sensitivity analysis for pooled disease-specific survival estimation.

Sensitivity analysis, in which 1 cohort was removed at a time and the pooled results were recalculated, was conducted to assess the stability of the results. No individual cohort significantly affected the overall HRs for OS (Fig. 5A) and DFS (Fig. 5B). Voduc KD's research significantly affected the pooled HRs for DSS, but the direction of the effect did not change (Fig. 5C). This finding shows that the results of this meta-analysis are credible.

**Figure 6:**
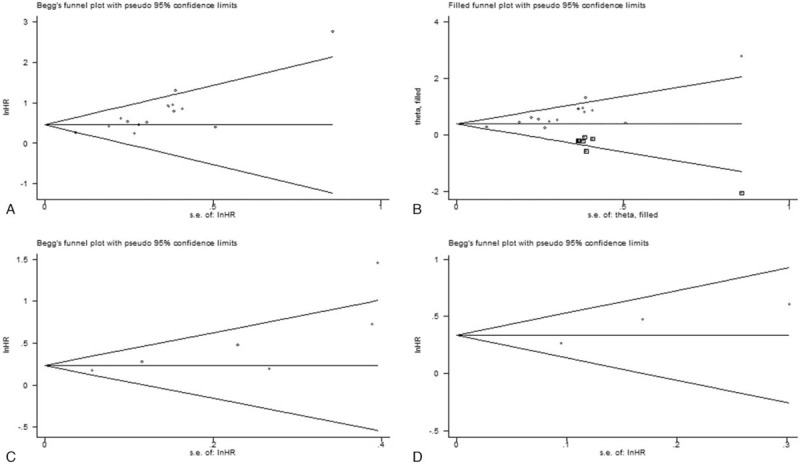
Begg funnel plots for assessment of potential publication bias in studies of CRYAB in patients with solid tumor. (A) Funnel plot of publication bias for overall survival. (B) Funnel plot adjusted with trim and fill methods for overall survival. (C) Funnel plot of publication bias for disease-free survival. (D) Funnel plot of publication bias for disease-specific survival.

In the meta-analysis of OS, publication bias was indicated by Begg test (*P* = .013), Egger test (*P* < .001) analysis, and the obvious asymmetric funnel plot (Fig. 6A). After adjusted by the trim and fill analysis, seven non-published studies were needed to balance the funnel plot (Fig. 6B), and the recalculated HR and 95% CI changed slightly (HR = 1.47, 95% CI: 1.20–1.80) but remained significant, indicating that the potential publication bias did not significantly affect the overall outcome. In the meta-analysis of DFS and DSS, no publication bias was observed as assessed using Begg tests (DFS: *P* = .060; DSS: *P* = .296), Egger tests (DFS: *P* = .052; DSS: *P* = .189), and funnel plots (DFS: Fig. 6C; DSS: Fig. 6D).

## 4 Discussion

High levels of CRYAB have been reported in a variety of human solid tumors and found to promote tumorigenesis and progression.^[26,27]^ Many clinical studies have been conducted on the prognostic value of CRYAB overexpression. However, most of these studies involved a limited number of patients and with inconclusive results. Therefore, we conducted this meta-analysis to determine the association between CRYAB expression and the prognosis of solid tumor patients.

This meta-analysis included 17 studies, including 18 cohorts with 6000 patients, and the systematically evaluated outcomes showed that high CRYAB level was significantly associated with poor OS, DFS, and DSS in various solid tumors. Moreover, sensitivity and publication bias analyses proved that the pooled results were stable. However, such a correlation was not found in a few subgroup analyses of OS. This finding was mainly attributed to the small sample size or the HR from the univariate analysis in 1 study of each subgroup. Moreover, high CRYAB expression was correlated with certain phenotypes of tumor aggressiveness, such as lymph node metastasis, distant metastasis, advanced clinical stage, low OS rate, and high recurrence rate. Thus, high CRYAB expression is associated with poor prognosis and certain phenotypes of tumor aggressiveness. The evaluation of CRYAB after tumor surgery may be a useful and easily available parameter that can be used to predict the outcome of treatment and select patients who require more aggressive treatment. The classification of patients with a high risk of poor prognosis based on CRYAB expression can lay the foundation for the introduction of targeted therapy, which may open up a new paradigm for cancer treatment. The basic biological mechanism behind the prognostic significance of CRYAB needs to be further evaluated.

CRYAB plays a vital role in inhibiting cell apoptosis that is 1 of the hallmarks of malignant diseases.^[28]^ CRYAB inhibits the autocatalytic maturation of caspase-3 and interacts directly with the proapoptotic Bcl-2 family proteins, such as Bax and Bcl-xs, to prevent mitochondrial translocation, thereby reducing cell apoptosis.^[29,30]^ Similarly, CRYAB interacts with p53 to block its translocation to the mitochondria, thereby indirectly inhibiting their proapoptotic effect on apoptotic Bcl-2 molecules.^[31]^ CRYAB inhibits the calcium-activated Raf/MEK/ERK signaling pathway-mediated p53-dependent apoptosis by inhibiting Ras activation.^[32]^ In addition, CRYAB participates in the regulation of intracellular apoptosis signals, which inhibit cell apoptosis by activating the Akt signaling pathway and enhancing phosphoinositide 3 kinase activity.^[33]^ Furthermore, tumor necrosis factor-related apoptosis inducing ligand can selectively induce apoptosis of cancer cells with almost no toxic effect on normal cells; thus, it has been tested as a promising anticancer agent.^[34,35]^ However, many cancer patients with high CRYAB expression are resistant to tumor necrosis factor-related apoptosis inducing ligand-induced apoptosis by reducing the tumor's sensitivity to cancer treatment.^[34,36]^ Therefore, CRYAB may serve as a new survival predictive biomarker and therapeutic molecule for cancer patients by participating in apoptosis.

Numerous studies have shown that high CRYAB expression in tumor tissues is closely related to invasion and metastasis. EMT is considered as a key regulator of cell invasion and metastasis in various cancers by conferring an aggressive phenotype. Shi et al demonstrated that CRYAB promotes the invasion and metastasis of colorectal cancer cells via EMT and is accompanied by a decrease in the expression of epithelial marker E-cadherin and an increase in the expression of mesenchymal markers.^[26]^ Besides, Chen et al, found that CRYAB contributes to gastric cancer cell migration and invasion via EMT, mediated by the nuclear factor-k-gene binding signaling pathway.^[37]^ CRYAB triggers EMT in cancer cells by activating the ERK signaling pathways.^[27,38]^ Furthermore, accumulating evidence indicated that CRYAB can enhance tumor angiogenesis, invasion, and metastasis by regulating the vascular endothelial growth factor.^[39,40]^ Thus, high CRYAB expression is closely related to cancer invasion and metastasis in solid tumor patients and may serve as a promising candidate biomarker for anti-cancer invasion and metastasis.

Although our meta-analysis indicated that high CRYAB expression is associated with poor prognosis, this work has several limitations that must be considered. First, a uniform criterion for the critical value of CRYAB expression is non-existent, and the cutoff value of each study is different, which may lead to bias in determining the role of CRYAB in tumor prognosis. Second, some HRs with 95% CIs were not directly provided in studies but estimated from Kaplan-Meier survival curves, which may have affected our results. Third, this study did not control anti-cancer treatment that has a certain influence on the survival time of cancer patients, which will affect the results. Fourth, the research region included in the article was mostly Asia, especially China, which affected the promotion of the results to a certain extent. Finally, the articles included in this meta-analysis were retrospective cohort studies, some of which had small sample sizes. Thus, better designed studies with larger sample sizes are needed to further confirm our results.

## 5 Conclusion

In summary, our results indicated that high CRYAB expression is associated with poor survival in patients with solid tumors and that CRYAB may be a promising prognostic biomarker and therapeutic target. We also found that CRYAB had significant prognostic value for OS in various cancer subgroups, such as digestive system cancers, head and neck cancer, OC, and NSCLC, whereas the prognostic impact of CRYAB was not statistically significant in BC and RCC. Therefore, larger-scale prospective studies using standardized methodologies are needed to assess the prognostic effect of CRYAB in BC and RCC.

## Author contributions

Feng Tian conceived of the idea and designed the study; Minglan Yang and Yufan Li searched, selected materials and extracted data; Minglan Yang analyzed the data; Minglan Yang and Feng Tian wrote the manuscript. All authors reviewed the manuscript.

**Conceptualization:** Feng Tian.

**Data curation:** Yufan Li.

**Investigation:** Minglan Yang, Yufan Li.

**Methodology:** Minglan Yang.

**Project administration:** Feng Tian.

**Software:** Minglan Yang.

**Supervision:** Feng Tian.

**Writing – original draft:** Minglan Yang, Feng Tian.

## References

[1] Bray F Ferlay J Soerjomataram I. Global cancer statistics 2018: GLOBOCAN estimates of incidence and mortality worldwide for 36 cancers in 185 countries. CA Cancer J Clin 2018;68:394–424. 30207593 10.3322/caac.21492

[2] Yu M Hao B Zhan Y. Krüppel-like factor 4 expression in solid tumor prognosis: a meta-analysis. Clin Chim Acta 2018;485:50–9. 29940144 10.1016/j.cca.2018.06.030

[3] Wang S Zeng J Xiao R. Poor prognosis and SATB1 overexpression in solid tumors: a meta-analysis. Cancer Manag Res 2018;10:1471–8. 29922091 10.2147/CMAR.S165497 PMC5997180

[4] Wang B Lu Z Huang Y. Prognostic impact of lncRNA-ATB expression in malignant solid tumors: a meta-analysis. Pathol Res Pract 2020;216:152897. 32146004 10.1016/j.prp.2020.152897

[5] Annertz K Enoksson J Williams R. Alpha B-crystallin – a validated prognostic factor for poor prognosis in squamous cell carcinoma of the oral cavity. Acta Otolaryngol 2014;134:543–50. 24702231 10.3109/00016489.2013.872293

[6] Tao X Cheng L Li Y. Expression of CRYAB with the angiogenesis and poor prognosis for human gastric cancer. Medicine 2019;98:e17799. 31702632 10.1097/MD.0000000000017799 PMC6855521

[7] Tan L Sha L Hou N. High ( B-crystallin and p53 co-expression is associated with poor prognosis in ovarian cancer. Bioscience Rep 2019;39. 10.1042/BSR20182407 PMC6579977 31152111

[8] Ousman SS Tomooka BH van Noort JM. Protective and therapeutic role for (B-crystallin in autoimmune demyelination. NATURE 2007;448:474–9. 17568699 10.1038/nature05935

[9] Zeng L Deng X Zhong J. Prognostic value of biomarkers EpCAM and (B-crystallin associated with lymphatic metastasis in breast cancer by iTRAQ analysis. BMC Cancer 2019;19:831. 31443698 10.1186/s12885-019-6016-3 PMC6708189

[10] Gu J Xu FK Zhu QL. The role and clinical significance of ( B—Crystallin in the proliferation and migration of non-small cell lung cancer. Fudan Univ J Med Sci 2018;45:323–9.

[11] Xu L Xu XY Lin ZZ. Differentiated expression and clinical significance of CRYAB in LSCC. Acta Universitatis Medicinalis Nanjing (Natural Science) 2016;36:1095–100.

[12] Kim MS Lee HW Jun SY. Expression of alpha B crystallin and BCL2 in patients with infiltrating ductal carcinoma. Int J Clin Exp Pathol 2015;8:8842–56. 26464626 PMC4583858

[13] Zhu J Feng Y Zhang F. Expression of alpha B-crystallin in colorectal cancer and its clinical significances. Chin J Bases Clin General Surg 2015;22:1332–7.

[14] Kim MS Lee HW Lee EH. Renal tumor with alpha B crystallin expression. Int J Clin Exp Pathol 2015;8:9383–9. 26464692 PMC4583924

[15] Voduc KD Nielsen TO Perou CM. (B-crystallin Expression in Breast Cancer is Associated with Brain Metastasis. NPJ breast cancer 2015;1:15014. 27656679 10.1038/npjbcancer.2015.14 PMC5027912

[16] Shi C He Z Hou N. Alpha B-crystallin correlates with poor survival in colorectal cancer. Int J Clin Exp Patho 2014;7:6056. PMC4203222 25337251

[17] Qin H Ni Y Tong J. Elevated expression of CRYAB predicts unfavorable prognosis in non-small cell lung cancer. Med Oncol 2014;31:142. 25048725 10.1007/s12032-014-0142-1

[18] Ho P Chueh S Chiou S. B-Crystallin in clear cell renal cell carcinoma: tumor progression and prognostic significance. Urol Oncol 2013;31:1367–77. 22417627 10.1016/j.urolonc.2012.01.015

[19] Mao Y Zhang DW Lin H. Alpha B-crystallin is a new prognostic marker for laryngeal squamous cell carcinoma. J Exp Clin Cancer Res 2012;31:101. 23231769 10.1186/1756-9966-31-101 PMC3551651

[20] Kim HS Lee Y Lim YA. B-Crystallin is a novel oncoprotein associated with poor prognosis in breast cancer. J Breast Cancer 2011;14:14. 21847389 10.4048/jbc.2011.14.1.14 PMC3148513

[21] Tang Q Liu Y Zhu X. Expression and prognostic significance of the B-crystallin gene in human hepatocellular carcinoma. Hum Pathol 2009;40:300–5. 18992912 10.1016/j.humpath.2008.09.002

[22] Moyano JV. B-Crystallin is a novel oncoprotein that predicts poor clinical outcome in breast cancer. J Clin Invest 2006;116:261–70. 16395408 10.1172/JCI25888 PMC1323258

[23] Stang A. Critical evaluation of the Newcastle-Ottawa scale for the assessment of the quality of nonrandomized studies in meta-analyses. Eur J Epidemiol 2010;25:603–5. 20652370 10.1007/s10654-010-9491-z

[24] Higgins JP Thompson SG Deeks JJ. Measuring inconsistency in meta-analyses. BMJ 2003;327:557–60. 12958120 10.1136/bmj.327.7414.557 PMC192859

[25] Egger M Smith GD Schneider M. Bias in meta-analysis detected by a simple, graphical test. BMJ 1997;315:629–34. 9310563 10.1136/bmj.315.7109.629 PMC2127453

[26] Shi C Yang X Bu X. Alpha B-crystallin promotes the invasion and metastasis of colorectal cancer via epithelial-mesenchymal transition. Biochem Biophys Res Commun 2017;489:369–74. 28506831 10.1016/j.bbrc.2017.05.070

[27] Li Q Wang Y Lai Y. HspB5 correlates with poor prognosis in colorectal cancer and prompts epithelial-mesenchymal transition through ERK signaling. PLOS One 2017;12:e182588. 10.1371/journal.pone.0182588 PMC5552184 28796798

[28] Mehlen P Kretz-Remy C Preville X. Human hsp27, Drosophila hsp27 and human alphaB-crystallin expression-mediated increase in glutathione is essential for the protective activity of these proteins against TNFalpha-induced cell death. EMBO J 1996;15:2695–706. 8654367 PMC450205

[29] Gruvberger-Saal SK. Is the small heat shock protein B-crystallin an oncogene? J Clin Invest 2005;116:30–2. 10.1172/JCI27462 PMC1323271 16395401

[30] Kamradt MC Chen F Cryns VL. The small heat shock protein B-Crystallin Negatively Regulates Cytochromec and caspase-8-dependent activation of caspase-3 by inhibiting its autoproteolytic maturation. J Biol Chem 2001;276:16059–63. 11274139 10.1074/jbc.C100107200

[31] Liu S Yan B Lai W. As a novel p53 direct target, bidirectional gene HspB2/(B-crystallin regulates the ROS level and Warburg effect. Biochimica et Biophysica Acta (BBA) - Gene Regulatory Mechanisms 2014;1839:592–603. 24859470 10.1016/j.bbagrm.2014.05.017

[32] Liu ES Raimann A Chae BT. c-Raf promotes angiogenesis during normal growth plate maturation. Development 2016;143:348–55. 26657770 10.1242/dev.127142 PMC4725343

[33] Zhang J Liu J Wu J. Progression of the role of CRYAB in signaling pathways and cancers. Onco Targets Ther 2019;12:4129–39. 31239701 10.2147/OTT.S201799 PMC6553995

[34] Volkmann J Reuning U Rudelius M. High expression of crystallin aB represents an independent molecular marker for unfavourable ovarian cancer patient outcome and impairs TRAIL- and cisplatin-induced apoptosis in human ovarian cancer cells. Int J Cancer 2013;132:2820–32. 23225306 10.1002/ijc.27975

[35] Duiker EW de Vries EGE Mahalingam D. Enhanced antitumor efficacy of a DR5-specific TRAIL variant over recombinant human TRAIL in a bioluminescent ovarian cancer xenograft model. Clin Cancer Res 2009;15:2048–57. 19276284 10.1158/1078-0432.CCR-08-1535

[36] Campbell-Lloyd AJM Mundy J Deva R. Is alpha-B crystallin an independent marker for prognosis in lung cancer? Heart Lung Circ 2013;22:759–66. 23582651 10.1016/j.hlc.2013.01.014

[37] Chen D Cao G Qiao C. Alpha B-crystallin promotes the invasion and metastasis of gastric cancer via NF-(B-induced epithelial-mesenchymal transition. J Cell Mol Med 2018;22:3215–22. 29566309 10.1111/jcmm.13602 PMC5980171

[38] Huang XY Ke AW Shi GM. alphaB-crystallin complexes with 14-3-3zeta to induce epithelial-mesenchymal transition and resistance to sorafenib in hepatocellular carcinoma. Hepatology 2013;57:2235–47. 23316005 10.1002/hep.26255

[39] Chen W Lu Q Lu L. Increased levels of alphaB-crystallin in vitreous fluid of patients with proliferative diabetic retinopathy and correlation with vascular endothelial growth factor. Clin Exp Ophthalmol 2017;45:379–84. 27928876 10.1111/ceo.12891

[40] Dong Z Kase S Ando R. Expression of (B-crystallin and vascular endothelial growth factor in conjunctival squamous cell carcinoma. Anticancer Res 2013;33:3745. 24023305

